# The need to improve access to rabies post-exposure vaccines: Lessons from Tanzania

**DOI:** 10.1016/j.vaccine.2018.08.086

**Published:** 2019-10-03

**Authors:** Joel Changalucha, Rachel Steenson, Eleanor Grieve, Sarah Cleaveland, Tiziana Lembo, Kennedy Lushasi, Geofrey Mchau, Zacharia Mtema, Maganga Sambo, Alphoncina Nanai, Nicodem J. Govella, Angel Dilip, Lwitiko Sikana, Francesco Ventura, Katie Hampson

**Affiliations:** a Ifakara Health Institute, P.O. Box 78373, Dar es Salaam, Tanzania; b Boyd Orr Centre for Population and Ecosystem Health, Institute of Biodiversity, Animal Health and Comparative Medicine, University of Glasgow, UK; c Health Economics and Health Technology Assessment (HEHTA), Institute of Health and Wellbeing, 1 Lilybank Gardens, University of Glasgow, UK; d Ministry of Health, Community Development, Gender, Elderly and Children, P.O. Box 573 Dodoma, Tanzania; e World Health Organization, Tanzania Country Office, P.O. Box 9292, Dar es Salaam, Tanzania

**Keywords:** Post-exposure prophylaxis, Dog-mediated rabies, Rabies prevention, Canine rabies, Immunoglobulin, Intradermal, Intramuscular, Vaccine regimen, Supply chain, Procurement

## Abstract

**Background:**

Rabies is preventable through prompt administration of post-exposure prophylaxis (PEP) to exposed persons, but PEP access is limited in many rabies-endemic countries. We investigated how access to PEP can be improved to better prevent human rabies.

**Methods:**

Using data from different settings in Tanzania, including contact tracing (2,367 probable rabies exposures identified) and large-scale mobile phone-based surveillance (24,999 patient records), we estimated the incidence of rabies exposures and bite-injuries, and examined health seeking and health outcomes in relation to PEP access. We used surveys and qualitative interviews with stakeholders within the health system to further characterise PEP supply and triangulate these findings.

**Results:**

Incidence of bite-injury patients was related to dog population sizes, with higher incidence in districts with lower human:dog ratios and urban centres. A substantial percentage (25%) of probable rabies exposures did not seek care due to costs and limited appreciation of risk. Upon seeking care a further 15% of probable rabies exposed persons did not obtain PEP due to shortages, cost barriers or misadvice. Of those that initiated PEP, 46% did not complete the course. If no PEP was administered, the risk of developing rabies following a probable rabies exposure was high (0.165), with bites to the head carrying most risk. Decentralized and free PEP increased the probability that patients received PEP and reduced delays in initiating PEP. No major difficulties were encountered by health workers whilst switching to dose-sparing ID administration of PEP. Health infrastructure also includes sufficient cold chain capacity to support improved PEP provision. However, high costs to governments and patients currently limits the supply chain and PEP access. The cost barrier was exacerbated by decentralization of budgets, with priority given to purchase of cheaper medicines for other conditions. Reactive procurement resulted in limited and unresponsive PEP supply, increasing costs and risks to bite victims.

**Conclusion:**

PEP access could be improved and rabies deaths reduced through ring-fenced procurement, switching to dose-sparing ID regimens and free provision of PEP.

## Highlights

• Prompt post-exposure vaccination is extremely effective in preventing human rabies.
• Intradermal post-exposure vaccination is easily adopted by health workers in Tanzania.
• High costs of PEP to government affect the supply chain and limit its availability.
• Limited PEP supply results in higher out-of-pocket payments and increased risks.
• Investment to facilitate free PEP provision would reduce rabies deaths.

## 1 Introduction

The burden of human rabies is high in many low- and middle-income countries (LMICs) where the disease is maintained and spread primarily by domestic dogs [1]. Following onset of symptoms, rabies is invariably fatal [2], however disease can be prevented in exposed persons through timely Post-Exposure Prophylaxis (PEP). The World Health Organization (WHO) recommended protocol for PEP includes immediate wound washing, administration of rabies vaccine and in severe exposures, infiltration of purified rabies immunoglobulin (RIG) into the wound(s) [1]. However PEP is expensive and costs can be a major obstacle to both bite victims and to local and national governments in LMICs [3], [4]. In some countries PEP is therefore bought only in relatively small amounts and distributed to a limited set of facilities. High out-of-pocket costs for bite victims paying for PEP (>$80/course) and limited availability can lead to delays in access, heightened rabies risks and even deaths, which are disproportionate in poor and marginalized communities [3]. Thus, although rabies is entirely preventable, limited access to PEP is thought to be a major reason why so many human rabies deaths continue to occur. Improving access to PEP for persons bitten by rabid animals is therefore crucial to achieving the goal of zero human deaths from dog-mediated rabies by 2030 [5].

There are a number of ways in which access to rabies PEP could be improved. For example, rabies vaccines can be administered to patients using dose-sparing intradermal (ID) regimens, which can generate substantial cost savings compared to intramuscular (IM) administration and reduce the occurrence of PEP stock-outs [6], [7]. Intradermal regimens have been adopted in several countries in Asia [8], but in most rabies endemic countries PEP is delivered via the IM route and there is little documentation on the feasibility and potential cost savings associated with ID administration in settings in Sub-Saharan Africa. Moreover, because PEP is needed in emergency situations, the supply chain must be responsive to this need. However rabies incidence is lower than other priority diseases in LMICs such as malaria, HIV and TB. Many persons exposed to rabies do not seek care and their clinical outcomes are unknown. A lack of diagnostic infrastructure means that when human rabies victims present to facilities showing neurological syndromes they are also often misdiagnosed [9]. The result is that health workers must administer a complicated vaccination regimen for a disease that they may not perceive to be common. Misdiagnosis, misadvise and underreporting confound uncertainties around the rabies burden and the potential impact of PEP. Frequent stockouts and long distances to major health facilities where PEP is available contribute to limited access [3]. Concerns remain about the operational feasibility of improving access to PEP including questions of supply chain management, cold chain and training needs, and on the effect of increased access to rabies PEP on demand and impact.

In recognition of these knowledge gaps, Gavi, the Vaccine Alliance, set up a learning agenda for rabies in 2013, to generate evidence on the programmatic impact and operational use of human rabies vaccines in endemic settings. We used data from studies across different settings in Tanzania to address these knowledge gaps, including interventions that aimed to improve access to PEP by reducing out-of-pocket costs to patients, decentralizing provision at peripheral health centres, and introducing ID vaccination. We synthesize lessons learned from these studies in Tanzania relating to the rabies burden, the PEP supply chain, health seeking and compliance by persons exposed to rabies and the efficacy of PEP.

## 2 Methods

We conducted studies across different settings in Tanzania including areas where interventions were undertaken to control rabies and prevent human deaths. We examined the incidence of rabies exposures and bites for which patients sought healthcare, and health seeking behaviours and health outcomes in relation to PEP access using two sources of data: contact tracing and mobile phone-based surveillance data. Specifically we conducted contact tracing in Serengeti and Ngorongoro districts in northern Tanzania (2002–2017) and in 14 selected districts in southern Tanzania (4 on Pemba island, and 10 in mainland Tanzania, grouping urban municipalities with corresponding rural districts; 2011–2017); and implemented a rabies specific mobile phone-based surveillance system across 28 districts in 7 regions of southern Tanzania (2011–2016) [10]. Access to PEP varied across these settings. Specifically, patients pay for PEP in most of Tanzania where it is typically only available from the district or regional hospital. This was the case in Serengeti and Ngorongoro districts in Northern Tanzania, but in the 28 districts in southern Tanzania PEP was provided for free through a WHO-coordinated rabies elimination demonstration project that began in 2010 and ended in 2015 [10], [11]. During this time PEP was supplied free-of-charge to hospitals and selected outlying facilities in each district and training was provided to over 300 health workers in use of the updated Thai Red Cross ID regimen [12] as a replacement to an IM regimen (d0, d7, d28) used elsewhere in Tanzania. We also conducted qualitative interviews with stakeholders at different levels within the health system to characterise the logistics associated with PEP provision and to triangulate our findings.

### 2.1 Data collection

Information on rabies exposures and bites for which patients sought healthcare, and health seeking behaviours and health outcomes in relation to PEP access was collected through contact tracing and mobile phone-based surveillance as described below.

In 2010 mobile phone-based surveillance was implemented across 28 districts in southern Tanzania to monitor the intervention undertaken to improve PEP access [10]. Mobile phones configured with a surveillance application were provided to participating facilities and health workers trained to report details of bite patients and PEP use on standardized forms on these phones. Mobile surveillance data was then collected routinely from January 2011 until November 2016 for these 28 districts, resulting in 24,999 records of patient presentations or 23,187 records after removal of records of patients who travelled from outside of the study districts.

Contact tracing began in 2002 in Serengeti and Ngorongoro districts in northern Tanzania and in the 11 districts in southern Tanzania in 2011 where mobile phone-based surveillance was also conducted. Hospital records of bite patients were used to initiate contact tracing following previously described methods [13]. Briefly, this involved exhaustive investigations to ascertain the status of the biting animal and identify additional bite victims who did not seek care. A biting animal was considered probable for rabies, according to WHO case definitions [14], if at least 2 clinical signs were evident and the animal died, was killed or disappeared within a 10 day period of the exposure. From tracing 5,168 patients who presented to health centres with bite injuries, we identified 2,367 who were exposed to bites by probable rabid animals (including 484 who did not report to a health centre). Where possible samples were collected from biting animals and cases confirmed either through field testing using a rapid diagnostic kit (Bionote, Korea), or by real-time PCR assay undertaken at the Animal and Plant Health Agency (APHA) in the UK [15]. The Bionote rapid diagnostic tests have been validated in field and laboratory settings [16], [17]. We used the PCR assay because it has been shown to be sensitive and specific even on degraded samples such as those sent from Tanzania, whereas the gold standard OIE tests FAT and RTCIT are sensitive to degradation [18], [19]. We previously validated our classification of animals as probable rabid according to WHO definitions using these tests, with 83% of samples from animals classified as probable for rabies testing positive by PCR (n = 313) and 90% using the rapid diagnostic kits (n = 175) [15].

A survey was used to describe the supply chain for PEP in Dodoma region in Tanzania where no interventions had been implemented to improve access. The survey was conducted from April to June 2016 targeting health workers responsible for requesting essential medicines in 92 public health facilities and 5 pharmacists responsible for managing these requests at district- and regional-level. In addition, between May and June 2017, purposively sampled stakeholders from the health system in Dodoma and Dar es Salaam regions were qualitatively interviewed about PEP management. These comprised 17 participants from both public and private facilities; 3 health workers responsible for administering PEP to bite victims from three health facilities; 3 pharmacists from these same facilities responsible for requesting PEP; 4 immunisation and vaccination officers (two at district-level and two at regional-level); 5 national officials from the Ministry of Health, Community Development, Gender, Elderly and Children (MoHCDGEC); and 2 pharmacists from private suppliers. The national officials included two from the parastatal health supply chain arm, the Medical Stores Department (MSD), involved in procurement and customer service; one from the Immunisation and Vaccination Department (IVD) and two from the Department of Preventive Services. Interviews were conducted in Swahili at participants’ place of work and took around 45 min each, covering the logistics of procurement, distribution, storage and use of PEP. Recordings were subsequently transcribed and translated into English.

### 2.2 Data analysis

#### 2.2.1 Incidence of bite-injury patients and probable rabies exposures

We used the mobile phone-based surveillance records from 2011 to 2016 to quantify the incidence of patients presenting to clinics in Southern Tanzania due to animal bites as well as clinic records used for contact tracing in Serengeti and Ngorongoro districts from 2003 to 2006 prior to district-wide dog vaccination campaigns, adjusting population sizes under district population growth rate projections [20]. We used a generalized linear mixed effects model (GLMM) to model bite patients per 100,000 at district-level, with setting (urban/rural) and estimated human:dog ratios [21] examined as predictors, and year and district as random effects. We also determined the average annual incidence of probable rabies exposures in Serengeti and Ngorongoro districts from contact tracing.

#### 2.2.2 Rabies risk and effectiveness of PEP

To estimate the protective effect of PEP we used a subset of the contact tracing data. Only individuals bitten by probable rabid animals were considered (n = 2,367), with individuals that received RIG (n = 1) or whose deaths were caused by tetanus or injury (n = 1 and 5, respectively) removed. We grouped exposures according to the part of the body where the person had been bitten. Individuals who did not provide details of bite location (n = 504) were excluded for this. For individuals with multiple bites (n = 290), only the highest risk bite was used for this categorization, established via the following hierarchy of risk (head > arms/hands > legs/feet > trunk) [22]. Based on the health outcomes of bite victims who did not receive PEP the probability of developing rabies following a bite to a specific body part was calculated. We subsequently re-checked the risk hierarchy and re-categorized exposures with the new hierarchy (head > trunk > arms/hands > legs/feet), re-calculating the probability of death according to bite site. Using these groupings we calculated the probability that in the absence of post-exposure vaccination a person would develop rabies given the site of the bite. By simulating from a mixture model, we estimated the probability of an exposed person developing rabies in the absence of PEP using these data on bite sites and infection.

Using the contact tracing data we assessed bite patients timeliness and completion of post-exposure vaccination (hereafter referred to as PEP, as RIG was only provided to a single patient in this study). We defined ‘timely’ PEP as initiated on the same day as the bite, and ‘late’ PEP as initiated more than 24 h after the bite. We considered 3 or more doses of PEP ‘complete’ and fewer than 3 doses ‘incomplete’. We then calculated the effectiveness of complete PEP and incomplete or late PEP in preventing rabies.

#### 2.2.3 Health seeking, PEP access and provision

We used contact tracing data to determine what proportion of bite patients presenting to clinics were due to probable rabid animals, animals of unknown status and healthy animals, and what proportion of probable rabies exposures sought and obtained PEP. From the contact tracing data we present the variability in patient delays to initiating PEP (n = 1,388). We also compare rates of completion of PEP between districts in northern Tanzania where patients pay for PEP (1,655 probable rabies exposures) and districts from southern Tanzania with free PEP (703 probable rabies exposures). We also assess variability in the number and location of facilities that patients visited for PEP, including the travel distance from their village (centroid) to the facility, from the mobile surveillance records in Southern Tanzania.

Interview data on PEP provision from health system stakeholders were coded using Framework analysis [23], guided by the topic questions, with inclusion of emerging themes. The framework approach outlined four levels of analysis: familiarisation with the data; identification of themes (forecasting, requisition and procurement, distribution, storage, monitoring, administration and reporting); coding interviewee responses to themes; and interpretation.

### 2.3 Ethical clearance

This work was approved by the Institutional Review Board of Ifakara Health Institute (IHI/IRB/No: 011-2016), the Medical Research Coordinating Committee of the National Institute for Medical Research of Tanzania (NIMR/HQ/R.8a/Vol.IX/946) and the University of Glasgow MVLS college ethics committee (200150148). A letter of approval to conduct interviews with key informants responsible for PEP was obtained from the MoHCDGEC on April 18, 2017.

## 3 Results

### 3.1 Incidence of bite-injury patients and probable rabies exposures

**Fig. 1:**
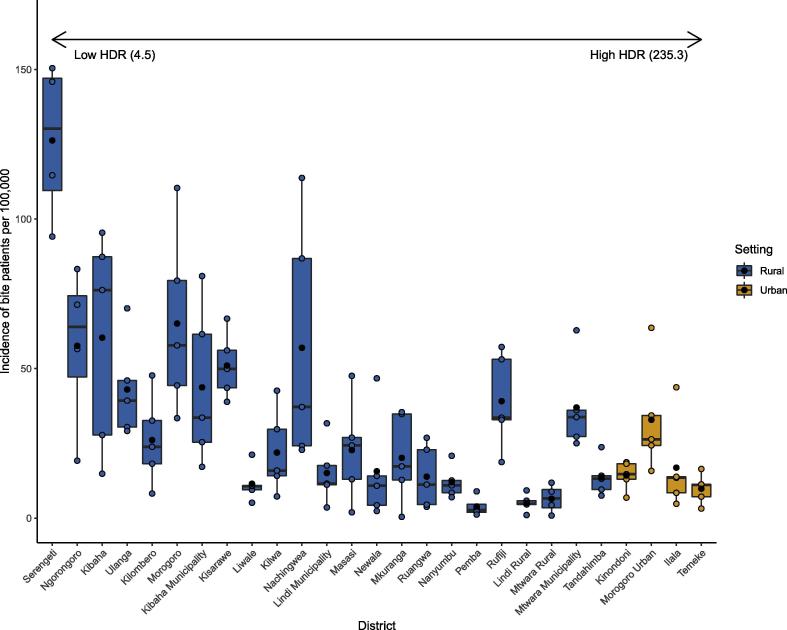
Variation in the annual incidence of patients presenting to clinics across Tanzania with bite injuries. Points are ordered by the estimated human:dog ratio for each district, and districts are coloured according to whether they are urban or rural. Data from twenty-eight districts in Southern Tanzania (2011–2016) are shown together with data from Serengeti and Ngorongoro (2003–2006, prior to routine annual dog vaccination campaigns). Black points show the average annual incidence, coloured points show annual data and the box and whiskers show the range and interquartile range.

The average incidence of bite injuries in patients presenting to health facilities varied considerably across Tanzania by district (Fig. 1), from less than 4 patients/100,000 persons per year to over 120/100,000. We found that bite-injury incidence was negatively correlated with human:dog ratios, i.e higher incidence in districts with more dogs per capita (regression coefficient: −25.298, p < 0.01, Std. Error: 8.220) and also higher in urban areas compared to rural areas once human:dog ratios were accounted for (coefficient: 47.56, p < 0.05, Std. Error: 22.634).

We detected an average of 75.6 and 19.3 probable rabies exposures per 100,000 persons per year in northern Tanzania from Serengeti and Ngorongoro districts, respectively, prior to the implementation of regular dog vaccination campaigns. These districts have low human:dog ratios (4.5 and 7, respectively) and bite victims must pay for PEP, as is routine in Tanzania. About 36% of patient presentations at health facilities were due to bites from probable rabid dogs (1,878/5,162 patients that sought care) as assessed through contact tracing, with the remainder from healthy animals or animals with unknown status. Around 25% of probable rabid dog bite victims identified through contact tracing in Serengeti and Ngorongoro did not seek care (418/1,655), and would therefore not be captured in health facility surveillance records (such as the mobile phone-based surveillance).

#### 3.1.1 Rabies risk and effectiveness of PEP

In the absence of PEP, we estimated that the risk of developing rabies from a probable rabid animal bite was 0.165 (95%CI 0.133–0.201), based on the proportion of victims bitten on different parts of the body and the risk of infection given the bite site (Table 1). Bites to the head and the neck carried the greatest risk of rabies (probability = 0.385, 95%CI 0.234–0.554), but bites to the trunk were also very high risk (probability = 0.215, 95%CI 0.123–0.335). From contact tracing we found that PEP administration was effective in preventing the onset of rabies in 473 patients exposed to probable rabid dogs who all promptly received PEP (vaccination within 1 day of the exposure, but no RIG) and completed the course (at least 3 doses). For this sample size, we estimate that prompt timely (adequate) post-exposure vaccination prevents rabies with probability 1.00 (95%CI 0.992–1.00). Of 1005 individuals identified during contact tracing who received late and/or incomplete post-exposure vaccination, 14 died showing clinical signs of rabies (Table 2), although none were laboratory confirmed. The probability of developing rabies under inadequate PEP (more than 1 day late and/or less than 3 doses) was calculated as 0.014 (95%CI 0.008–0.023), i.e. inadequate PEP prevents rabies with probability 0.986 (95% CI 0.977–0.992). Nine of these deaths were attributable to delays in PEP administration, and five to initiation of post-exposure vaccination without delay but completion of only 1–2 doses (Table 2).

**Table 1:** Probable rabies exposures and deaths according to the site of the body where bitten and whether PEP was administered. Overall probability of death was estimated from a mixture model given the locations on the body where people were bitten.

Bite location	Probability of death (95% CI)	Number of deaths	Probable rabies exposures that did not receive PEP	Probability of bite depending on location on body	Number of probable rabies exposures
Head	0.385 (0.234–0.554)	15	39	0.088 (0.076–0.102)	164
Trunk	0.215 (0.123–0.335)	14	65	0.138 (0.122–0.155)	258
Arm/hands	0.141 (0.086–0.213)	18	128	0.315 (0.293–0.335)	586
Leg/feet	0.127 (0.087–0.176)	30	237	0.459 (0.436–0.481)	855
Overall	0.165 (0.133–0.201)	77	469	–	1863

**Table 2:** Details of human rabies deaths where some form of PEP was received. Two bite victims received 4 doses of vaccine but in both cases there were delays in administering PEP (4 days and 5 days, respectively). One patient developed rabies after prompt vaccination, but completed only 2 vaccine doses. IM = Intramuscular, ID = Intradermal.

PEP Failure	Age (years)	Sex	Doses received	Route of PEP	Days till PEP	Number of wounds	Location of Bite(s)
Delayed	14	Male	4	IM	5	1	Arm
Delayed	3	Female	4	IM	4	1	Hand
Delayed	3	Male	3	IM	1	1	Head
Delayed & Incomplete	6	Female	2	IM	10	1	Hand
Delayed & Incomplete	16	Male	2	IM	6	2	Arm, Hand
Delayed & Incomplete	7	Male	2	IM	1	3	Head, Hand, Trunk
Delayed & Incomplete	8	Female	2	IM	1	2	Head, Hand
Incomplete	11	Male	2	ID	0	2	Head, Trunk
Delayed & Incomplete	5	Male	1	IM	3	1	Hand
Delayed & Incomplete	21	Male	1	IM	1	1	Head
Incomplete	85	Male	1	ID	0	1	Leg
Incomplete	8	Male	1	ID	0	2	Head, Foot
Incomplete	9	Male	1	ID	0	1	Head
Incomplete	70	Female	1	ID	0	4	Arm, Hand, Trunk, Leg

### 3.2 Health seeking, PEP access and provision

Throughout most of Tanzania, PEP is still administered following a 3-dose IM schedule (d0, d7, d28) [4], despite national guidelines being updated in 2013 and 2017 to recommend the 5-dose Essen IM regimen and the 4-dose Updated Thai Red Cross ID regimen [24], [25]. Typically patients must also pay for PEP. However, in districts where the WHO-coordinated rabies elimination project took place, the updated Thai Red Cross ID schedule is now used routinely. ID administration was introduced to designated health workers responsible for PEP as part of the project during a one-day workshop [11]. On-job training in the mobile phone-based surveillance system, including further instruction in ID vaccination was completed, taking around 3 h at each facility [10], [11]. No major difficulties were encountered in switching to ID administration, however health workers reported occasional shortages of needles for ID use [11]. RIG use in Tanzania is negligible; none of the interviewed health workers reported ever using RIG, except for those from Dar es Salaam who were only aware of RIG from the WHO-coordinated project.

Bite patients are first required to consult a clinician for wound assessment, including payment of a consultation fee, before being referred for PEP to the Reproductive and Child Health (RCH) unit, which provides vaccination services. RCH units open Monday through Friday 8 am to 2 pm, with patients reporting out of hours required to wait till the RCH opens. In Dar es Salaam, bite victims should present a letter from the veterinary department before receiving PEP. The purpose of these letters is to help health workers ascertain the health status of the biting animal and to enable livestock officers to follow up the biting animal in case of an outbreak. However, few bite victims complete this process and livestock officers rarely investigate biting animals.

In Tanzania payment procedures for PEP differ by facility; some health workers collect payment directly, while some patients pay at the facility cashier before vaccination. Only a small proportion of patients pay via health insurance (<10% of Tanzanian citizens have insurance) [26]. Exemption to vulnerable groups and waivers to poor bite victims were reported during stakeholder interviews, but PEP was generally not considered to be part of exemption services. The cost charged for PEP typically included a marginal profit to enable facilities to continue stocking it, at the discretion of district authorities. Most facilities including private pharmacies charge around 30,000 Tanzania shillings ($13) for a 1 ml or 0.5 ml vaccine vial. Participants from the main health centre in Dar es Salaam reported now charging per clinic visit at 15,000 TSh (∼$7) for 2 × 0.1 ml intradermal injections, recovering around 60,000–75,000 TSh (∼$27–33) per vial. Some patients also purchase vaccine at private pharmacies and bring these to RCH units for administration.

**Fig. 2:**
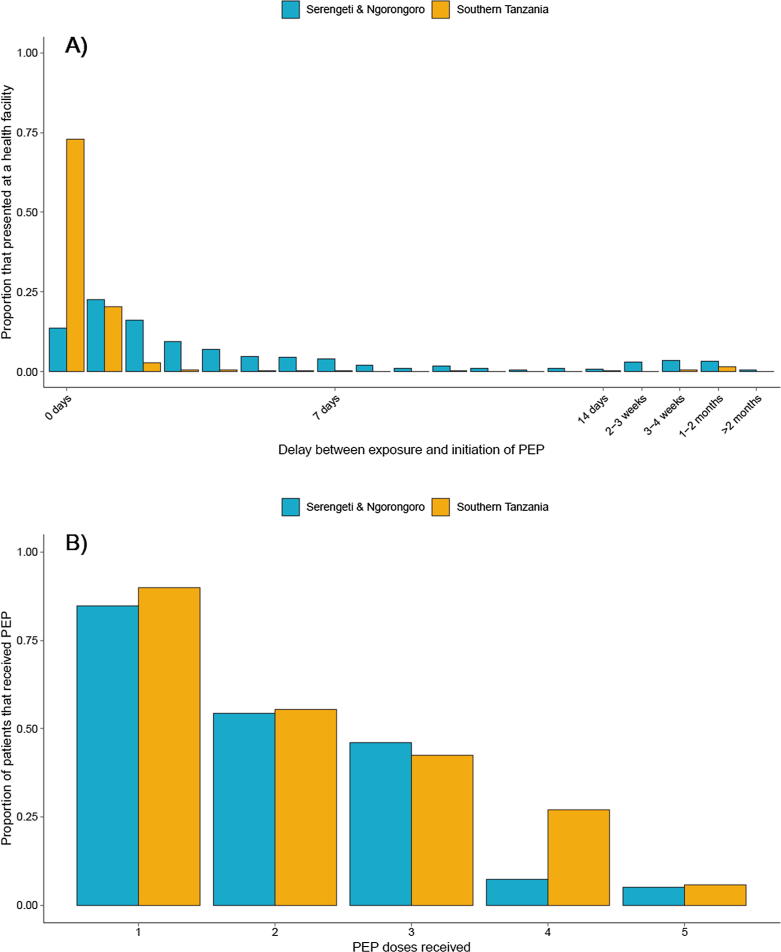
Initiation and completion of post-exposure vaccination according to access: (A) delay between date bitten and first post-exposure vaccination for individuals bitten by probable rabid animals and (B) proportion of patients that received 1–5 doses of PEP. Blue indicates locations where patients were required to pay for PEP (Serengeti and Ngorongoro) and yellow indicates locations where PEP was provided for free (28 districts in Southern Tanzania). Panel A shows contact tracing data on delays between exposure and initiation of PEP for rabies exposed persons (781 exposures from Serengeti and Ngorongoro districts, and 607 exposures from 11 districts in southern Tanzania). Out of 794 patients identified through contact tracing who had delayed PEP (more than 1 day late), nine deaths occurred (Table 2). Panel B shows mobile phone-based surveillance records from Southern Tanzania (yellow, n = 21,692) of PEP completion and contact tracing data on rabies exposed patients from Serengeti and Ngorongoro districts (n = 1,200). Not all bite victims received the first PEP dose because of shortages at the facility or costs required to purchase PEP. Rabies exposed persons who did not seek care (identified through contact tracing) are not shown. In Serengeti district patients were typically vaccinated following a 3 dose IM regimen (d0, d7, d28) and in Ngorongoro district following the 5-dose Essen IM regimen (d0, d3, d7, d14, d28). In southern Tanzania, most patients were vaccinated following the updated Thai Red Cross ID regimen (d0, d3, d7, d28).

In Serengeti and Ngorongoro district where patients pay for PEP, 25.3% (418/1,655) of probable rabies exposed persons did not seek care, citing both costs and lack of awareness about rabies. On seeking care a further 15.3% (189/1,237) still did not initiate PEP due to costs, shortages or occasionally incorrect advice (usually at peripheral centres that should refer patients). Delays to administration of PEP were also common. Most probable rabies exposed persons who initiated PEP did so on the same day as exposure (Fig. 2A). However, in northern Tanzania, where patients pay for PEP, the median delay for those that initiated PEP was 2 days, whereas in Southern Tanzania, where PEP was provided for free, the median delay to initiating PEP was zero days (Fig. 2A). Some rabies exposed persons reported to health facilities only after a delay of several months, sometimes only after another person bitten by the same animal showed signs of rabies. Delays were often conflated with non-completion, as a consequence of barriers to access, including costs and limited availability from only a few facilities. Decentralized and free supply of PEP, as implemented in the 28 districts in southern Tanzania from 2010 onwards [11], was associated with a higher probability of patients initiating PEP (0.899 versus 0.873 in Serengeti and Ngorongoro; Fig. 2B). However, around 6% (1,458/23,150) of these patients in southern Tanzania also faced shortages, although they might have obtained PEP subsequently at another facility. A relatively low proportion of patients completed at least 3 doses of PEP (47.3% of patients from mobile surveillance records in southern Tanzania versus 54.2% of rabies exposed bite victims, identified through contact tracing, who initiated PEP, Fig. 2B).

**Fig. 3:**
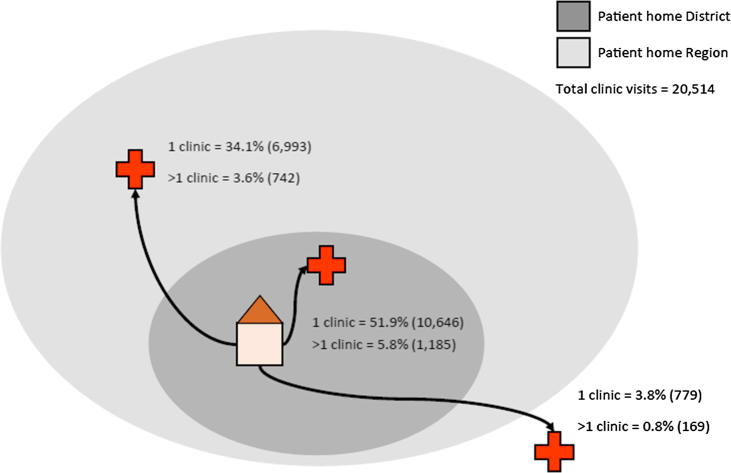
Schematic of the number of visits to health facilities made by patients to obtain PEP within their district, in other district in their region, and in other regions. All clinic visits were from patients located within the study districts. The schematic also shows the proportion of visits made to multiple facilities and their locations.

In the districts in southern Tanzania with improved PEP access, 89.8% (18,418/20,514 excluding records where health facility name was not recorded) of patients obtained PEP from a single health facility, with the remainder seeking and obtaining PEP at multiple (up to 4) health facilities. About 37.7% (7,735/20,514) travelled outside of their home district to receive PEP, and 4.6% (948/20,514) travelled to another region (Fig. 3). Interviewees reported that PEP access had declined throughout Tanzania after the Ministry of Health discontinued centralized PEP procurement in 2012 and issued a directive that instead local government authorities allocate funds for PEP. Areas covered by the WHO-coordinated rabies elimination demonstration project only experienced this decline after the project ended in 2015. Bite victims were reported to often travel from other regions where PEP is not available in either public or private clinics. From the mobile surveillance records 7.25% (1,812/24,999) of patients originated from outside of the study districts where PEP was provided for free (these patients were not included in all other reported statistics). Patients from districts in southern Tanzania travelled an average distance (as the crow flies) of 30 km (95%CI 1–116 km) from their home to a health facility (range: 0–673 km). If all health facilities had provided PEP, this travel distance could have been reduced to 12 km (95% CI 1–37 km).

Both rabies vaccines and RIG are listed as essential medicines that are supplied by MSD to public health facilities through the Tanzanian Integrated Logistics System (ILS) [27], and to private facilities on request. However, although classified for administration at hospitals and health centers [24], PEP is not listed among the 251 predetermined priority medicines in the ILS requisition forms, bought from the allocated MOH budget for quarterly supply. As a consequence, PEP is procured through district funds obtained from other sources, for example basket funds (pooled donor and government funds to support the implementation of the Health Sector Strategic Plan IV) and funds recovered from health insurance or out-of-pocket payments. One pharmacist stated “*the money is not enough, that is why we normally say let's just get this money from this source and buy*” (respondent 3). Because of this atypical and unpredictable financing, PEP is usually procured outside the MSD distribution calendar, therefore facilities normally arrange collection themselves. Private suppliers are used to source medical supplies when MSD is out of stock. During stockouts, MSD issues an out-of-stock form to allow facilities to purchase PEP from private suppliers and all requests are directed to specific pre-qualified private suppliers (denoted prime vendors). Some private retailers reported preferring to source cheaper PEP because the price from some suppliers was unaffordable for bite victims, but this also led to stocking delays. Interviewees stated that PEP stock-outs were common, usually lasting for around two months, although sometimes for shorter periods (days to weeks). Delays in recovering funds were reported to contribute to stockouts, as some private suppliers refused to resupply health facilities with standing debts.

There is no PEP forecasting at district or regional level, so purchase is only made once stocks are depleted. At MSD, customers are required to show the source of their revenue in relation to their demand, therefore the amount of PEP procured depends on funds in hand even if costs are expected to be recovered through, for example, insurance. Both MSD and health facilities only procure PEP if they can guarantee cost recovery. The resulting supply chain is therefore not responsive to fluctuations in PEP demand, such as during outbreaks when presenting patients exceed available stock. As reported by one pharmacist “…*..the forecast is difficult because the patients are unpredictable* …*. after a long period of vaccine shortage, the other health facilities stopped providing services, and you will see all burden coming to us*” (respondent 1).

The cold chain system was considered by interviewees to be satisfactory, with every district and region equipped with large refrigerators, and most health facilities (>90%) with small refrigerators. However, these refrigerators are prioritized to store vaccines by the Immunisation and Vaccination Development department (formerly EPI). Health workers were concerned that criteria used for evaluating health facility performance disincentivize storage of PEP. In practice PEP are usually stored with routine vaccines but participants suggested guidelines should be revised “…*..One of the things which they do in the routine vaccine to assess the performance of a facility in storage, is on assessing if their fridge is not keeping other vaccine other than the routine vaccine.* …*..credits are deducted for those facilities found keeping other vaccine”* (respondent 16).

No standard tools are used for tracking PEP use and bite victims are not issued with vaccination cards as for routine vaccination. Instead health workers use registers to record bite victims. These differ by facility but usually include the patient address, bite site and PEP dose. The infectious disease weekly ending (IDWE) report is used by MOH for reporting bite patients to national level (whether or not they obtained PEP) on a weekly basis.

## 4 Discussion

We show that although post-exposure vaccination is highly effective in preventing rabies, even in the absence of RIG, costs to patients and governments are a major obstacle limiting access in Tanzania. These high costs directly affect how PEP is distributed, with PEP only procured reactively and in limited quantities. Out-of-pocket payments increase if patients need to travel to multiple clinics due to stockouts. The risk of developing rabies is very high for rabies exposed patients who do not obtain PEP and also increase with delays to PEP initiation and noncompletion. Nonetheless, an effective cold chain and demonstrated success in switching to ID administration highlight opportunities for improving PEP access, especially if the primary cost barrier could be overcome through free provision, like other immunisation services.

Access to PEP is limited in many countries with endemic rabies and PEP is often only available from health facilities in the capital city [28]. Patients also generally need to pay for PEP and these costs are reported as a major obstacle for many bite victims. In Tanzania health policy recognizes immunisation as a free service [29], but bite victims are required to pay for PEP and a concerning proportion do not initiate PEP or are delayed because of costs (both direct and indirect) [3], [30]. Although health insurance reduces out-of-pocket payments for healthcare [31], less than 10% of Tanzanians are insured [26]. Exemption and waivers to cost-sharing occasionally enable poor bite victims to obtain PEP for free, but procedures are cumbersome and inefficient [32], which may also delay initiating PEP. Even though PEP costs are borne largely by bite victims, high costs to local and national governments further limits availability and results in frequent stockouts. Other essential medicines are prioritized, leaving insufficient funds for PEP procurement. Although ILS was designed as an efficient platform for essential medicine delivery [27], various limitations render it ineffective for PEP [33] and district resource allocation tools (CCHP, IFMS and EPICOR systems) disadvantage PEP compared to other essential medicines [34], [35].

In Tanzania, the incidence of bite injuries requiring PEP is highly variable. The size of dog populations influences incidence; in districts with many dogs (low human:dog ratios) bite-injuries are correspondingly higher. A large proportion of these bite patients are probable rabies exposures who urgently require PEP. The high costs of PEP likely explain the high proportion of bite patients due to rabid dogs in Tanzania, as persons with non-severe bites from evidently healthy dogs may be less likely to seek treatment, whereas those bitten severely and by high risk dogs generally seek care. In settings in Southeast Asia where PEP is subsidized, health seeking can be much higher [28], [34], [35], [36], [37]. In these settings, although a much smaller proportion of bites are attributed to probable rabid animals compared to our study sites in Tanzania, PEP is often administered also in the event of healthy animal bites. If PEP were to be provided for free in Tanzania, training in integrated bite case management could limit unnecessary PEP administration to persons bitten by healthy or vaccinated animals [38], [39]. Knowledge of the size of dog populations could also inform PEP allocation, but the supply chain needs to be responsive, which requires the use of tools for tracking PEP use.

Experiences from southern Tanzania highlighted opportunities for improving access to PEP and reducing rabies deaths. Provision of PEP free-of-charge improved health seeking [10], [11], while switching to dose-sparing ID regimens required only minimal training [10], [11]. During a recent shortage of PEP in Dodoma region, researchers were able to facilitate access by obtaining vaccine from another region and training clinicians in ID administration to enable more patients to be treated with the limited supply. More generally, training for health workers could facilitate adoption of the latest WHO recommendations for accelerated PEP that aims to reduce direct and indirect costs to bite victims [1]. Further training would also be necessary if tools were introduced to track PEP use. The wide coverage of an effective cold chain system [40], strengthened through EPI, also provides infrastructure for PEP storage, although guidelines need updating to incentivise safe storage of PEP. Similarly, RCH units need to better accommodate PEP scheduling to ensure that patients can receive these life-saving vaccines without delay. Although the current supply chain for PEP in Tanzania has major limitations, the efficiency of vertical programs in controlling TB and HIV/AIDS in many LMICs [41], including EPI for childhood immunizations, demonstrate that alternative supply systems can be effectively implemented, when medicines are considered a public good and universal health coverage is promoted.

We have brought together diverse data on the incidence of rabies exposures and bite patients, and on healthcare utilization and provision across different parts of Tanzania. However, not all of these data are directly comparable, given differences in dog population sizes, dog vaccination effort and provision of health services. The probability that a bite patient receives PEP is likely to be overestimated based on stockouts reported from mobile surveillance records, as patients may subsequently seek and obtain PEP elsewhere. Hence the benefits of free PEP provision may not be fully captured from health records that do not longitudinally track individual health seeking outcomes. In contrast estimates from contact tracing should capture these aspects of health seeking, but may be more subject to inaccuracies in bite victim recall. Contact tracing itself may also increase PEP compliance as bite victims are advised regarding essential rabies prevention strategies during interviews. We also report the effectiveness of PEP in preventing rabies based on exposures from probable but not confirmed rabid dogs. However, we have found high correspondence between probable rabies cases identified on the basis of their clinical history and on subsequent laboratory confirmation (83–90%) [15].

## 5 Conclusion

A large number of preventable deaths from rabies occur in Tanzania due to poor access to PEP. We conclude that free provision of PEP at point-of-care, ring-fenced PEP procurement, switching to recommended dose-sparing ID regimens, and ensuring responsive and accountable supply chains for PEP are all feasible approaches that should reduce the burden of human rabies.
